# HLA-B*58 and HLA-C*2 Alleles Are Associated with the Occurrence of Rheumatoid Arthritis Among Omanis

**DOI:** 10.3390/jcm14041219

**Published:** 2025-02-13

**Authors:** Mohammed S. Al-Balushi, Irin Antony, Ali H. Al-Shirawi, Hamad Al-Riyami, Jumaa Z. Al-Busaidi, Crystal Y. Koh, Khalid M. Al-Naamani, Sidgi S. Hasson, Ali A. Al-Jabri, Elias A. Said

**Affiliations:** 1 Department of Microbiology and Immunology, College of Medicine and Health Sciences, Sultan Qaboos University, P.O. Box 35, Muscat 123, Omanaaljabri@squ.edu.om (A.A.A.-J.); 2 Department of Medicine, College of Medicine and Health Sciences, Sultan Qaboos University, P.O. Box 35, Muscat 123, Oman; 3 Department of Genetics, College of Medicine and Health Sciences, Sultan Qaboos University, P.O. Box 35, Muscat 123, Oman; 4 Department of Medicine, The Medical City for Military and Security Services, P.O. Box 35, Muscat 123, Oman; noumani73@gmail.com

**Keywords:** rheumatoid arthritis, HLA, autoimmune, CD8 T cells, NK cells

## Abstract

**Background/Objectives:** Rheumatoid arthritis (RA) is an autoimmune disease that is influenced by polymorphisms in the HLA molecules. Only a few studies assessed the presence of an association between HLA class I genes and RA. Moreover, ethnic background influences the association of HLA molecules and RA. HLA-I molecules are essential for the activation of CD8 T cells and natural killer (NK) cells. The implication of these cells in RA pathogenesis is controversial. Therefore, we investigated the presence of associations between HLA-I alleles and RA in Omani patients. **Methods**: HLA class I alleles were genotyped in a total of 206 volunteers (102 RA patients and 104 controls). The control group included volunteers who were not affected by any known disease. The Chi square test was used to investigate the significance of the associations between the HLA alleles and the occurrence of RA. A corrected *p* value (*p*_c_) was calculated using the Bonferroni correction. **Results**: The frequency of HLA-B*58 was ≈2.7-fold lower in RA patients (10.8%) compared to the control group (28.8%; *p*_c_ = 0.0324). Moreover, the frequency of HLA-C*02 in RA patient was ≈8-fold higher compared to the control group (*p*_c_ = 0.0104). **Conclusions**: This study is the first to demonstrate the presence of association between HLA-B*58 and HLA-C*02 and the occurrence of RA, which could guide future research on targeted therapies. It also suggests that these HLA alleles might influence CD8 T cells and NK cells implication in RA pathogenesis.

## 1. Introduction

Rheumatoid arthritis (RA) is a chronic systemic inflammatory autoimmune disease that affects 0.3–1% of the human population [1]. Genetic factors contribute to 50–60% of the risk of developing RA [2]. These factors can be divided into genes related to the major histocompatibility complex (*MHC*) region and genes outside the *MHC* [non-human leukocyte antigen (*HLA*)] [3]. HLA genes are highly polymorphic and their frequency and sequences are different among the different human populations [3,4]. The presence or absence of a particular HLA allele determines whether the potential autoantigen is presented and influences the response to that autoantigen [5].

The association of HLA molecules with RA was investigated in different human populations. HLA class II genes were found to be the most strongly associated HLA molecules with the development of RA, especially the shared epitope in HLA-DRB1 alleles [3]. However, only a few studies described the presence of associations between RA and HLA class I genes. The single nucleotide polymorphisms (SNPs) in the HLA B locus at amino acid position 9, which is located in the peptide binding groove, were found to be associated with RA [6]. Additionally, studies investigating the association of HLA-B*08 (Asp9) with RA, highlighted the importance of the 8.1 haplotype, which contains HLA-B*08 on an HLA-DRB1*03 background [7]. Furthermore, a recent study has reported that the majority of individuals with the HLA-B27/44 and B44/44 genotypes were diagnosed with definitive rheumatic disease [8]. Moreover, different HLA-C alleles, including HLA-C*07:04, HLA-C*w0303, HLA-C*w04, and HLA-Cw07 were also reported to be associated with RA [9,10].

HLA-A and -B are essential elements in the specific activation of CD8 T cells, whereas HLA-C plays an important role in modulating NK cell activation. The implication of CD8 T cells and NK cells in the pathogenesis of RA is controversial and poorly understood [10,11,12]. Therefore, investigating the presence of potential associations between HLA-I and RA can potentially provide elements that indicate the implication of CD8 T Cells and NK cells in RA pathogenesis.

Altogether a few studies assessed the association between HLA-I alleles and RA, and such association was never investigated in the Omani population, although the ethnic background may influence the presence of such association [3]. Therefore, in this study we investigated the presence of associations between HLA-I alleles and RA in Omani patients.

## 2. Material and Methods

### 2.1. Study Population

Blood samples were collected from 102 volunteer RA patients (Table 1) attending the Rheumatology Clinic at the Sultan Qaboos University Hospital (SQUH), a tertiary care hospital that accepts referral from the entire country. This ample represents about 1–1.6% of Omani patients with RA. RA patients fulfilled the 2010 American College of Rheumatology/European League Against Rheumatism (ACR/EULAR) classification criteria for RA. The control group included volunteers who were not affected by RA or any other known disease including autoimmune disease (*n* = 104; Table 1). They were all Omani and had the same average of age and same sex ratio as the control group. A total of 5ml blood was collected from each volunteer in tubes containing EDTA. Samples were stored at −20 °C until DNA extraction. The information on the participating patients was retrieved from the Hospital Information System (HIS) of Sultan Qaboos University Hospital (SQUH). The study was approved by the Ethics Committee of the College of Medicine and Health Sciences at the Sultan Qaboos University (MREC # 849). An informed consent form was signed by each volunteer.

### 2.2. DNA Isolation

Stored blood samples were thawed and equilibrated to room temperature to extract the DNA. DNA isolation was performed by using QIAamp DNA Blood Midi kit (QIAGEN, Germantown, MD, USA) following the manufacturer instructions. In brief, blood samples were processed by mixing with protease, Buffer AL, and ethanol, followed by incubation at 70 °C. The mixture was loaded onto a QIAamp Midi column, washed with Buffers AW1 and AW2, and centrifuged. Finally, DNA was eluted with 200 μL of AE buffer. The concentration and purity of the isolated DNA were checked using the Nanodrop (Thermo Scientific, Waltham, MA, USA).

### 2.3. HLA Typing

HLA class I alleles were genotyped using OLERUP HLA screening kits (Olerup, West Chester, PA, USA) by sequence specific primer (SSP)-polymerase chain reaction (PCR). PCR amplification of HLA class I (A, B, C) alleles was performed following the manufacturer protocol. The PCR products were loaded into a 1.3% agarose gel. The bands were visualized under the ultraviolet (UV_light. The pattern of the gel lanes with the specific PCR products was matched with the information in the lot-specific interpretation and entered in the SCORE software Version 5 of the OLERUP to identify the HLA alleles.

### 2.4. Statistical Analysis

The Chi square test was used to investigate the significance of the associations between the HLA alleles and the occurrence of RA. A corrected *p* value (*p*_c_) was calculated using the Bonferroni correction. This was performed by multiplying the calculated *p* value with the number of tested alleles for each locus [13]. A *p* value < 0.05 was considered statistically significant. The Statistical Package for Social Sciences (SPSS version 21), Microsoft Excel, and the websites https://www.icalcu.com/stat/chisqtest.html, (accessed on 14 June 2017) and https://www.socscistatistics.com/tests/chisquare/default2.aspx, (accessed on 14 June 2017) were used for data processing and analysis. Arlequin version 3 was used for allele frequency calculation.

## 3. Results

### 3.1. Absence of Association Between HLA-A Alleles and RA

**Figure 1:**
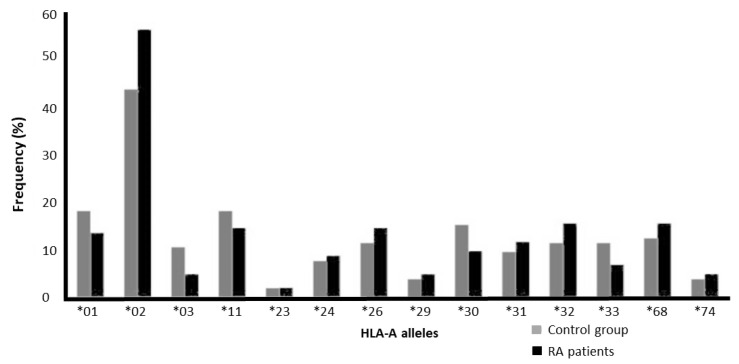
Frequency of HLA-A alleles among the control group and RA patients.

Our results showed that HLA-A*02 was the most frequent HLA-A allele among the control group (44.2%) and RA patients (56.9%). HLA-A*23 was the least frequent HLA-A in both groups (1.9% in the control group and 2% in RA patients). Figure 1 and Table 2 show the frequency of HLA-A alleles in RA patients and the control group. There was no HLA-A allele that was found only in the control group or the RA patients, and there was no significant difference in the frequencies of the HLA-A alleles between the groups (Table 2).

### 3.2. The Association Between HLA-B Alleles and RA

**Figure 2:**
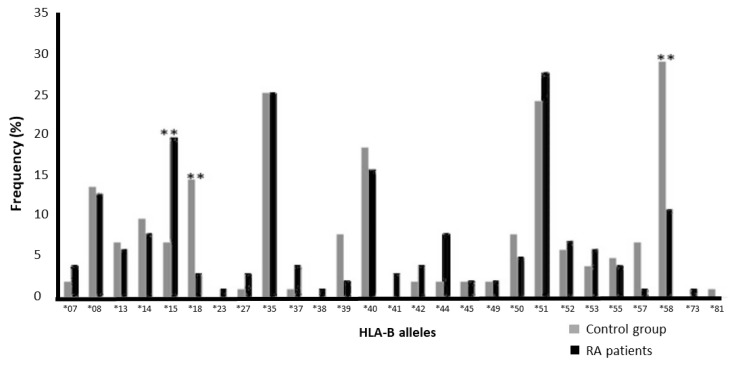
Frequency of HLA-B alleles among the control group and RA patients (** *p* < 0.01).

We found that HLA-B*58 was the most frequent HLA-B allele in the control group (28.8%), while HLA-B*51 was the most common HLA-B allele in the RA group (27.5%). HLA-B*27, HLA-B*37, and HLA-B*81 frequencies were the lowest in the control group (1%). The frequencies of HLA- B*23, HLA-B*38, HLA-B*57, and HLA-B*73 were the lowest in the RA patients (1%). HLA-B41* was present in three RA patients and absent in the control group (Figure 2 and Table 3). HLA-B*23, *38, and *73 were present in one RA patient only and not found in the control group (Figure 2 and Table 3). In contrast, HLA-B*81 was present in one donor in the control group and not observed in the RA patients (Figure 2 and Table 3). However, none of these alleles showed a significant association (Table 3).

The frequency of HLA-B*15 in the RA group (19.6%) was found to be ≈3-fold higher than that in the control group (6.7%; *p* = 0.007; Table 2). The frequency of HLA-B*18 in the patient group (2.9%) was found to be ≈5-fold lower than that in the control group (14.4; *p* = 0.005; Table 3). However, none of these associations remained significant when we applied the Bonferroni correction calculated by multiplying the calculated *p* value with the number of tested alleles for this locus [13] (*p*_c_ = 0.189 and 0.135 for the alleles HLA-B*15 and *18, respectively). Moreover, the frequency of HLA-B*58 was found to be ≈2.7-fold lower in the patient group (10.8%) than in the control group (28.8%; *p* = 0.0012; Table 3). This association remained significant after applying the Bonferroni correction (*p*_c_ = 0.0324).

### 3.3. The Association of HLA-C Alleles with RA

**Figure 3:**
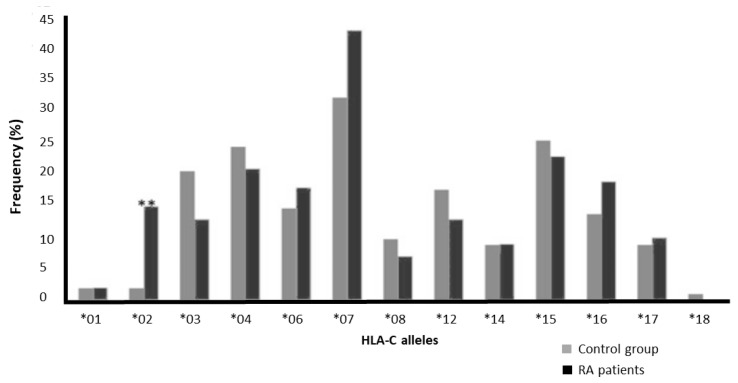
Frequency of HLA-C alleles among the control group and RA patients (** *p* < 0.01).

We observed that HLA-C*07 was the most frequent HLA-C allele in the control group (31.7%) and RA patients (42.2%). The frequency of HLA-C*18 was the lowest in the control group (1%). HLA-C*18 was present in one donor in the control group and not found in the RA patients (Figure 3 and Table 4). However, this allele did not show any significant association (Table 3). HLA-C*01 had the lowest values in RA patients (2%). Figure 3 and Table 3 show the frequency of HLA-C alleles in RA patients and the control group.

The frequency of HLA-C*02 in the RA group was found to be ≈8-fold higher than that in the control group (*p* = 0.0008; Table 4). This association remained significant after applying the Bonferroni correction calculated by multiplying the calculated *p* value with the number of tested alleles for this locus [13] (*p*_c_ = 0.0104).

## 4. Discussion

This study is the first to investigate the association of HLA-I polymorphisms with RA in the Omani population. Our results suggest that the presence of HLA-B*58 and HLA-C*02 alleles may have an impact on the occurrence of RA.

We found a significant association between HLA-B*58 and the protection against RA among Omanis. To our knowledge this association was not reported before. HLA-B is highly polymorphic and differences in the peptide presentation might contribute to the protective association observed with HLA-B [14]. The crystal structure of the central peptide residue of an antigenic peptide loaded onto some HLA-B*58 alleles might have either a buried or an exposed conformation in the peptide-binding groove. This can affect TCR recognition [15]. This suggests that HLA-B58 may have a lower capacity to activate autoreactive CD8 T cells that are specific for RA-related autoantigens. CD8 T cells may have a role in the stimulation of the immune response during RA, as CD8 T cells present in the synovial fluid can produce inflammatory cytokines such as TNF-α, which increases bone degradation by activating osteoclasts and other immune cells [12]. Therefore, this suggests that HLA-B58’s capacity to stimulate CD8 T cells might be low to a degree that limits the participation of CD8 T cells in the onset of RA. Other studies from the United States of America (USA) also reported that HLA-B is a risk locus for RA [6,7]. Together with our findings, these reports suggest that HLA-B alleles might influence the implication of CD8 T cells in the pathogenesis of RA. A recent study performed in the USA used a single cell transcriptome and CD8 T cell receptor (TCR) sequencing of CD8 to suggest that cytotoxic CD8+ T cells targeting citrullinated antigens play a role in driving synovitis and joint tissue destruction in anti-citrullinated protein antibodies (ACPA)-positive rheumatoid arthritis [8].

Our study also showed a significant association between HLA-C*02 and the susceptibility to RA in Omani patients. No previous study reported such association. HLA-C molecules can regulate NK cells activity in positive and negative manners [11]. HLA-C*02 can lead to the activation of NK cells through its interaction with KIR2DL1 [16] and it has the capacity to inhibit these cells as well by interacting with KIR2DS1 [17]. The role of NK cells in RA was previously reported, however it remains elusive. NK cells are present in the synovial fluid of RA patients [10,11]. NK cells may have a destructive effect during RA as they can be a source of TNF-α and IFN-γ, provide help to T and B cells and stimulate monocytes [10,11]. However, NK cells can also play a tolerogenic role during RA by blocking Th17 T cells, killing activated macrophages and inhibiting the differentiation of osteoclasts [11]. Therefore, polymorphisms in HLA-C*02 alleles might affect the regulation of NK cells during RA through its interaction with KIR2DS1 leading to a destructive effect for these cells. Other studies have also reported the presence of association between HLA-C*03, *04, *07, and *15 and the occurrence of RA [10,18,19]. A recent study from the United Kingdom (UK) demonstrated that CD8^+^CD57^+^KIR2DL1^+^ NK cells are associated with sustained remission [20]. Therefore, the results of these studies together with our results suggest that HLA-C alleles expressed in RA patients might influence the implication of NK cells in RA pathogenesis. Future studies should utilize high-resolution HLA genotyping alongside KIR receptor analysis to explore potential KIR-HLA interactions in RA. This approach will address current limitations and provide deeper insights into RA pathogenesis.

Future research should explore the molecular mechanisms behind these associations, HLA-B*58 and HLA-C*02 and the occurrence of RA. Experimental validation of the impact of HLA-B58 and HLA-C02 alleles on CD8+ T cell and NK cell functions could be achieved by conducting in vitro functional assays using cells from individuals with these alleles, examining cytokine production, cytotoxic activity, and antigen-specific responses [21,22]. Future studies can also incorporate KIR receptor analysis alongside HLA genotyping to gain deeper insights into the mechanisms of NK cell regulation.

We did not find any association between HLA-A alleles and RA. This might be related to the sample size in our study; however, to our knowledge, no other study documented such a relation, which supports our findings.

Many environmental factors contribute to RA pathogenesis. Cigarette smoking is a major factor, which has been found repeatedly in RA susceptibility. Other factors such as use of oral contraceptives, breast feeding, and dietary factors have only weak association with RA [23]. Such incremental factors were very likely similar between the control and RA group, due to the general culture, reducing the likelihood of bias.

## 5. Conclusions

This is the first study to show an association between HLA-B*58 and the protection from RA and between HLA-C*02 and the risk of RA occurrence. Therefore, HLA-B and HLA-C polymorphisms can potentially influence the occurrence of RA. Our findings provide novel insights into the genetic factors contributing to RA development in this population, potentially informing future diagnostic and therapeutic strategies.

## 6. Limitations of the Study

The limited sample size restricts the generalizability of the results to broader populations, warranting caution in interpreting the conclusions. Moreover, the study is conducted at a single center, which could introduce geographic bias and limit the diversity of the patient population. Including more participants from different regions of Oman in future studies would increase the statistical power of the analysis and allow for more reliable generalizations. However, as the center serves as a national referral hospital, the patient pool represents a broader demographic, somewhat mitigating this limitation. Furthermore, our data remain highly valid, as the studied population, representing 1 to 1.6% of the total patients, provides a statistically significant sample that reflects broader population trends. Moreover, the study utilizes standard HLA genotyping, which, due to its low resolution, may have missed detailed allelic variations; therefore, performing high-resolution HLA genotyping in future studies is crucial for uncovering precise allele variations and their potential associations, potentially yielding deeper insights into disease mechanisms and interactions.

## Figures and Tables

**Table 1:** Study population.

Study Population	Total (*n*)	Female/Male	Age
**RA patient group**	102	85/17	43 ± 16.7 (15–88)
**Control group**	104	87/17	42.9 ± 16.9 (15–96)

**Table 2:** Frequency of HLA-A alleles among the control group and RA patients.

Alleles in HLA-A Locus	Control (104)		Patients (102)		Exact *p* Value	RR	95% CI	Control (104)	Patients (102)
	No	%	No	%				Allele Frequency	Allele Frequency
A*01	19	18.3	14	13.7	0.449	0.712	0.336–1.510	0.111	0.078
A*02	46	44.2	58	56.9	0.072	1.662	0.958–2.883	0.26	0.328
A*03	11	10.6	5	4.9	0.192	0.436	0.146–1.302	0.053	0.029
A*11	19	18.3	15	14.7	0.575	0.771	0.368–1.617	0.096	0.074
A*23	2	1.9	2	2	1	1.02	0.141–7.382	0.01	0.01
A*24	8	7.7	9	8.8	0.805	1.161	0.430–3.138	0.043	0.049
A*26	12	11.5	15	14.7	0.541	1.322	0.586–2.982	0.067	0.074
A*29	4	3.8	5	4.9	0.747	1.289	0.336–4.942	0.019	0.025
A*30	16	15.4	10	9.8	0.295	0.598	0.257–1.388	0.082	0.049
A*31	10	9.6	12	11.8	0.658	1.253	0.516–3.045	0.048	0.059
A*32	12	11.5	16	15.7	0.421	1.426	0.638–3.188	0.063	0.088
A*33	12	11.5	7	6.9	0.336	0.565	0.213–1.498	0.058	0.034
A*68	13	12.5	16	15.7	0.552	1.302	0.592–2.867	0.072	0.078
A*74	4	3.8	5	4.9	0.747	1.289	0.336–4.942	0.019	0.025

**Table 3:** Frequency of HLA-B alleles among the control group and RA patients.

Alleles HLA-B Locus	Control (104)		Patients (102)		Exact *p* Value	RR	95% CI	Control (104)	Patients (102)
	No	%	No	%				Allele Frequency	Allele Frequency
B*07	2	1.9	4	3.9	0.443	2.082	0.373–11.623	0.01	0.02
B*08	14	13.5	13	12.7	1	0.939	0.418–2.110	0.067	0.074
B*13	7	6.7	6	5.9	1	0.866	0.281–2.671	0.034	0.029
B*14	10	9.6	8	7.8	0.806	0.8	0.302–2.006	0.048	0.039
B*15	7	6.7	20	19.6	0.007	3.38	1.361–8.393	0.038	0.103
B*18	15	14.4	3	2.9	0.005	0.18	0.050–0.642	0.072	0.015
B*23	0	0	1	1	0.495	1.01	0.991–1.030	0	0.005
B*27	1	1	3	2.9	0.366	3.121	0.319–30.513	0.005	0.015
B*35	26	25	26	25	1	1.026	0.547–1.925	0.12	0.127
B*37	1	1	4	3.9	0.21	4.204	0.462–38.273	0	0.02
B*38	0	0	1	1	0.495	1.01	0.991–1.030	0.005	0.005
B*39	8	7.7	2	2	0.101	0.24	0.050–1.159	0.038	0.01
B*40	19	18.3	16	15.7	0.712	0.832	0.401–1.726	0.091	0.093
B*41	0	0	3	2.9	0.12	1.03	0.996–1.066	0	0.015
B*42	2	1.9	4	3.9	0.443	2.082	0.373–11.623	0.01	0.025
B*44	2	1.9	8	7.8	0.057	4.34	0.899–20.960	0.01	0.039
B*45	2	1.9	2	2	1	1.02	0.141–7.382	0.01	0.01
B*49	2	1.9	2	2	1	1.02	0.141–7.382	0.01	0.01
B*50	8	7.7	5	4.9	0.569	0.619	0.195–1.958	0.038	0.025
B*51	25	24	28	27.5	0.634	1.196	0.640–2.235	0.13	0.167
B*52	6	5.8	7	6.9	0.782	1.204	0.390–3.712	0.029	0.039
B*53	4	3.8	6	5.9	0.536	1.563	0.428–5.709	0.019	0.029
B*55	5	4.8	4	3.9	1	0.808	0.211–3.099	0.024	0.02
B*57	7	6.7	1	1	0.065	0.137	0.017–1.136	0.034	0.005
B*58	30	28.8	11	10.8	0.002	0.298	0.140–0.635	0.144	0.054
B*73	0	0	1	1	0.495	1.01	0.991–1.030	0	0.005
B*81	1	1	0	0	1	0.99	0.972–1.09	0.005	0

**Table 4:** Frequency of HLA-C alleles among the control group and RA patients.

Alleles in HLA-C Locus	Control (104)		Patients (102)		Exact *p* Value	RR	95% CI	Control (104)	Patients (102)
	No	%	No	%				Allele Frequency	Allele Frequency
C*01	2	1.9	2	2	1	1.02	0.141–7.382	0.01	0.01
C*02	2	1.9	15	15	0.001	8.79	1.956–39.522	0.01	0.074
C*03	21	20	13	13	0.189	0.58	0.272–1.227	0.13	0.064
C*04	25	24	21	21	0.617	0.82	0.424–1.582	0.149	0.103
C*06	15	14	18	18	0.572	1.27	0.602–2.684	0.072	0.093
C*07	33	32	43	42	0.149	1.57	0.887–2.773	0.178	0.24
C*08	10	9.6	7	6.9	0.614	0.69	0.253–1.896	0.048	0.039
C*12	18	17	13	13	0.437	0.7	0.322–1.511	0.087	0.069
C*14	9	8.7	9	8.8	1	1.02	0.388–2.687	0.058	0.044
C*15	26	25	23	23	0.744	0.87	0.459–1.660	0.139	0.118
C*16	14	14	19	19	0.346	1.47	0.694–3.122	0.067	0.093
C*17	9	8.7	10	9.8	0.814	1.15	0.446–2.952	0.048	0.049
C*18	1	1	0	0	1	0.99	0.972–1.009	0.005	0

## Data Availability

The original contributions presented in this study are included in the article. Further inquiries can be directed to the corresponding authors.
